# The effect of gD-derived peptides on T cell immune response mediated by BTLA-HVEM protein complex in melanoma patients

**DOI:** 10.3389/fimmu.2024.1362152

**Published:** 2024-05-21

**Authors:** Karolina Wojciechowicz, Katarzyna Kuncewicz, Jacek Rutkowski, Jacek Jassem, Anna Wardowska, Marta Spodzieja

**Affiliations:** ^1^ Department of Physiopathology, Faculty of Medicine, Medical University of Gdańsk, Gdańsk, Poland; ^2^ Department of Biomedical Chemistry, Faculty of Chemistry, University of Gdańsk, Gdańsk, Poland; ^3^ Department of Oncology and Radiotherapy, Medical University of Gdańsk, Gdańsk, Poland

**Keywords:** immune checkpoints, BTLA-HVEM complex, melanoma, T cells, gD-derived peptide

## Abstract

**Introduction:**

The effector function of T cells is regulated via immune checkpoints, activating or inhibiting the immune response. The BTLA-HVEM complex, the inhibitory immune checkpoint, may act as one of the tumor immune escape mechanisms. Therefore, interfering with the binding of these proteins can prove beneficial in cancer treatment. Our study focused on peptides interacting with HVEM at the same place as BTLA, thus disrupting the BTLA-HVEM interaction. These peptides’ structure and amino acid sequences are based on the gD protein, the ligand of HVEM. Here, we investigated their immunomodulatory potential in melanoma patients.

**Methods:**

Flow cytometry analyses of activation, proliferation, and apoptosis of T cells from patients were performed. Additionally, we evaluated changes within the T cell memory compartment.

**Results:**

The most promising compound – Pep(2), increased the percentages of activated T cells and promoted their proliferation. Additionally, this peptide affected the proliferation rate and apoptosis of melanoma cell line in co-culture with T cells.

**Discussion:**

We conclude that the examined peptide may act as a booster for the immune system. Moreover, the adjuvant and activating properties of the gD-derived peptide could be used in a combinatory therapy with currently used ICI-based treatment. Our studies also demonstrate that even slight differences in the amino acid sequence of peptides and any changes in the position of the disulfide bond can strongly affect the immunomodulatory properties of compounds.

## 1 Introduction

High rates of cancer incidence and mortality constitute one of the biggest health issues worldwide. Cancer management is complex, tedious, and often unsuccessful due to unpredicted disease onset, propagation, and patient treatment response. The remarkable progress in cancer research unveiled several specific mechanisms behind neoplasm progression, and tumor immunosurveillance escape (1). Through the formation of a tumor microenvironment (TME), cancer cells are capable of maintaining proliferative signaling and evading growth suppressors, thus resisting cell death and gaining replicative immortality. Tumor-associated angiogenesis serves not only as a nutrient supply but also facilitates tumor invasion and metastasis (2). One of the ubiquitously used mechanisms of immune escape is linked to the upregulation of inhibitory immune checkpoints (ICPs) on tumor cells (1, 3, 4).

Immune checkpoints constitute a group of co-signaling molecules regulating immune responses crucial for T cell activation. The ICPs can be divided into two groups: stimulatory or inhibitory molecules. As their main function is to maintain immune system homeostasis, prevent autoimmunity, and sustain the ability to fight infections, ICPs have become an attractive target in immunomodulating therapies. The overexpression of various ICPs, including programmed death protein-1 (PD-1) and cytotoxic T lymphocyte antigen-4 (CTLA-4) on T cells and their ligands (PD-L1 and CD80/86, respectively) on cancer cells, is associated with an uncontrolled growth of tumor cells and immune system idleness. The introduction of immune checkpoint inhibitors (ICIs), i.e., anti-PD-1, anti-PD-L1, and anti-CTLA-4 antibodies, has revolutionized cancer treatment, providing unprecedented survival in some patients. Despite promising activity, currently used ICIs are not flawless, as they can induce a wide range of adverse effects, including autoaggression or acquired resistance to immunotherapy (5, 6). Moreover, the treatment response rate is still far from clinical expectations. Thus, the new immune pathways are in clinical consideration.

The BTLA-HVEM complex is another, yet not fully explored, inhibitory ICP with plausible application in cancer immunotherapy. The receptor, B- and T-lymphocyte attenuator (BTLA), is expressed on B and T lymphocytes, monocytes, macrophages, dendritic cells, and natural killer cells. Naive human T cells highly express BTLA but this expression gradually decreases upon activation. Upregulated expression of BTLA on CD8+ and CD4+ T cells in the TME was associated with unfavorable prognoses for patients with various types of cancer (7, 8). The ligand, herpes virus entry mediator (HVEM), acts as a two-edged sword because it can induce opposite cellular effects dependent on the interacting molecule. Being highly expressed on T and B cells, HVEM can interact either with lymphotoxin α (LTα) and LIGHT, providing a costimulatory signal, or with BTLA or cluster of differentiation 160 (CD160), leading to the suppression of the immune response (9). Moreover, HVEM interacts with herpes simplex virus-1 and -2 (HSV-1 and HSV-2) glycoprotein D (gD) (10, 11) and synaptic adhesion-like molecule 5 (SALM5) (12). HVEM is believed to be an element of tumor immune evasion, as its strong expression on malignant cells can mediate functional inhibition of BTLA+ T cells (4, 13–18). Several studies discussed the potential role of tumor cell-intrinsic BTLA-HVEM in tumor-mediated immunosuppression (7, 19). They suggested targeting this complex with its inhibitors to reverse tumor-induced T cell dysfunction (4). Several clinical trials have already addressed this issue and evaluate the BTLA-HVEM complex as a therapeutic target or biomarker in various malignancies (NCT04137900, NCT04278859, NCT04773951).

The first identified binding partner for HVEM was the glycoprotein D interacting with HVEM at the same site as BTLA. Moreover, it was shown that gD excludes BTLA from the complex with HVEM, enhancing the costimulatory HVEM-LIGHT pathway and increasing immune responses (20–23). We used the amino acid sequence of a *N*-terminal fragment of gD binding to HVEM (24) to design peptides targeting HVEM. Our previous reports describe the design, synthesis, and biochemical characteristics of several peptides based on the gD binding fragment. The four of them were selected for further biological evaluation, displaying the best potential as inhibitors of protein complex formation. We confirmed these peptides’ interaction with HVEM, using surface plasmon resonance (SPR) and their ability to inhibit the BTLA-HVEM complex formation using enzyme-linked immunosorbent assays (ELISA) and cellular assays (25, 26). We also confirmed that these molecules block the BTLA-HVEM interaction, not affecting the HVEM-LIGHT binding. Therefore, the peptides disrupt the inhibitory function of HVEM while its stimulatory role is preserved. Moreover, we performed biological studies on healthy human T cells to evaluate the immunomodulatory potential of the tested compounds. Based on the obtained results, we reported a visible impact of the peptides, especially Pep(2), on the activation state and proliferation of T cells (27).

In this study, we reported the influence of four gD-derived peptides on the activity of T cells obtained from melanoma patients (MP). The flow cytometry allowed for an extensive analysis of the activation state, proliferation rate, apoptosis, and the T cell memory compartment of MP T cells exposed to the examined peptides. The levels of cytokines, chemokines, and growth factors were also measured in the culture supernatants. All experiments were performed on a full range of peripheral blood mononuclear cells (PBMC) to warrant natural cell-to-cell interactions. Additionally, we studied the proliferation of melanoma cell line (SK-MEL-30) co-cultured with magnetically isolated healthy T cells treated with the examined peptides. Moreover, we compared peptides to evaluate whether slight differences in the amino acid sequence and the position of the disulfide bond could affect their biological activity. Herein, we show that even minor modifications greatly affect the immunomodulatory properties of the peptides. Based on the obtained results, we postulate that one of the gD-derived peptides, Pep(2), can restore T cell activity via disruption of the BTLA-HVEM interaction (25, 26).

## 2 Material and methods

### 2.1 The examined peptides

The four examined peptides are based on the *N*-terminal fragment of gD protein interacting with HVEM (**Table 1**, **Supplementary Table 1**). To design these peptides, we used the crystal structure of the HVEM-gD complex (PDB code: 1JMA) and performed the molecular mechanics generalized Born surface area (MM/GBSA) analysis for the protein complex. The *N*-terminal fragment of a native gD protein is disordered, but during the interaction with HVEM, it forms a β-hairpin structure. To enable the formation of a similar β-hairpin structure, we introduced disulfide bonds to the structure of synthesized gD-derived peptides. The design, synthesis, and chemical properties have previously been described (25, 26). In short, these peptides were synthesized by solid phase peptide synthesis (SPPS), purified by reverse phase-high performance liquid chromatography (RP-HPLC), then subjected to oxidation, and purified again. All these molecules interact with the HVEM protein, as confirmed using SPR, and disrupt the BTLA-HVEM complex formation in ELISA and cellular assays. These four peptides were chosen from a larger group of compounds due to their most promising inhibitory properties toward BTLA-HVEM complex formation (25, 26). Additionally, based on our previous studies, we selected the most effective concentration for the immunological evaluation (25, 26).

**Table 1:** The amino acid sequences of the peptides.

No.	Peptide name	Amino acid sequence
**Pep (1)**	gD(1-36) (K10C-T29C)	Ac-KYALVDASLC(&)MADPNRFRGKDLPVLDQLC(&)DPPGVRR-NH_2_
**Pep (2)**	gD(1-36) (K10C-D30C)	Ac-KYALVDASLC(&)MADPNRFRGKDLPVLDQLTC(&)PPGVRR-NH_2_
**Pep (3)**	gD(1-36) (A12C-L25C)	Ac-KYALVDASLKMC(&)DPNRFRGKDLPVC(&)DQLTDPPGVRR-NH_2_
**Pep (4)**	gD(1-38) (L4C-V37C)	Ac-KYAC(&)VDASLKMADPNRFRGKDLPVLDQLTDPPGVRRC(&)Y-NH_2_

### 2.2 Cell line and culture condition

The human melanoma cell line SK-MEL-30 was purchased from the Leibniz Institute DSMZ-German Collection of Microorganisms and Cell Cultures GmbH (DSMZ no.: ACC 151). The SK-MEL-30 cells were cultured in RPMI 1640 (Sigma Aldrich, Germany, #R8758) supplemented with 20% fetal bovine serum (FBS) (Sigma-Aldrich, Germany, #F9665), 100 μg/ml streptomycin, 100 U/ml penicillin (Sigma-Aldrich, Germany, #P0781) and 2 mM L-glutamine (Sigma-Aldrich, Germany, #G7513) at 37°C in 5% CO_2_. SK-MEL-30 cells, as adherent cells growing in monolayers, were enzymatically dissociated using 5ml of 0.25% trypsin-EDTA solution (Sigma-Aldrich, Germany, #T4049) prior to each passage. Cells were collected and subjected to the co-cultures with healthy donors (HD) T cells when the confluency reached 70–80%.

### 2.3 Melanoma patients

Treatment naive patients (n=10) diagnosed with advanced skin melanoma (MP – melanoma patients) were assigned for enrolment during their routine pre-treatment visit to the Department of Oncology and Radiotherapy, Medical University of Gdańsk, Poland. Blood samples were collected, provided the participant’s written informed consent. The study was approved by the Bioethics Committee for Scientific Research at the Medical University of Gdańsk (approval no. NKBBN/96–340/2022). All the procedures were performed by the principles of the Declaration of Helsinki.

The following inclusion criteria were applied: age >18 years, stage IIIB to IV pathologically confirmed melanoma (according to the 8th edition of the American Joint Committee on Cancer classification system) (28, 29), and Eastern Cooperative Oncology Group (ECOG) performance status 0–1 (30). To reduce the risk of immune system disruption the exclusion criteria were as follows: ongoing or prior cancer immunotherapy, immunosuppressive treatment (glucocorticosteroids, cytostatic, biological treatment), concomitant diseases significantly affecting the immune system, such as autoimmune connective tissue disease (currently or previously treated), active allergies and asthma (requiring any immunosuppression), heart attack and other severe cardiovascular incidents in the last 6 months; severe (GOLD stage 3 and 4) chronic obstructive pulmonary disease, poorly controlled diabetes, a history of organ/bone marrow transplantation, or any immunodeficiency or other active malignancy.

### 2.4 Peripheral blood mononuclear cell and magnetic T cell isolation

The MP blood samples obtained in the form of the whole blood collected in K3 EDTA vacutainer tubes or buffy coats (from HD) underwent density-gradient centrifugation with the usage of Histopaque®-1077 (Sigma-Aldrich, Germany, #10771) (by the manufacturer’s instructions) to obtain PBMCs. These cells were either used for proper tests or were magnetically separated with EasySep™ Human T Cell Isolation Kit (StemCell Technologies, USA, #17952). The magnetically isolated HD CD3+ T cells were immediately ready for co-cultures with the SK-MEL-30 cell line.

### 2.5 PBMC incubation with gD-derived peptides

PBMC obtained from MPs were stained with 1 µl Violet Proliferation Dye 450 (VPD450) (Becton Dickinson, USA, #562158) for 10–15 min in the dark at 37°C according to the manufacturer’s protocol and resuspended in a complete culture medium (RPMI 1640 culture medium supplemented with 10% FBS, 2 mM L-glutamine, 100 U/ml penicillin, and 100 μg/ml streptomycin). Then, cells were seeded into a 24-well plate at the density of 1.5 × 10^6^ cells/well. They were stimulated with the ImmunoCult™ Human CD3/CD28 T Cell Activator (Thermo Fisher Scientific, USA, #10971) in the presence of the examined peptides at the previously selected concentration (**Supplementary Table 1**). The cells were then incubated for 3 days in standard culture conditions (37°C, 5% CO_2_). Subsequently, cells were subjected to cytometric phenotypic analysis and an evaluation of proliferation and apoptosis. Two groups of cells were analyzed: (i) control (CTRL+): PBMC stimulated with CD3/CD28 mAb, and (ii) tested cells: PBMC stimulated with CD3/CD28 mAb and incubated with the examined peptides. Supernatants from PBMC cultures were collected for further cytokine profile evaluation.

### 2.6 Flow cytometry analysis of stimulated cultured cells

Flow cytometric analysis was used to evaluate the phenotype and activation status of MP PBMCs cultured with CD3/CD28 mAb and the examined peptides. For this purpose, all cultured cells were collected and stained with specific monoclonal antibodies after 72h incubation. The following antibodies were used: anti-CD3-FITC (Becton Dickinson, USA, #555332), anti-CD4-PerCP (Becton Dickinson, USA, #345770), anti-CD8-APC-H7 (Becton Dickinson, USA, #560179), anti-CD25-APC (Invitrogen, USA, #17-0259-42), anti-CD69-PE (Invitrogen, USA, #12-0699-42), anti-CD197-PE (Becton Dickinson, USA, #560765), anti-CD45RA-FITC (Becton Dickinson, USA, #555488), anti-BTLA-PE (Becton Dickinson, USA, #558485), anti-HVEM-AF647 (Becton Dickinson, USA, #564411).

The proliferation rates of stimulated PBMC and SK-MEL-30 cell were evaluated using the dividing cell tracking (DCT) method (30). Briefly, at the time of cell division, the VPD450 was distributed in half to the daughter cells, resulting in a 50% reduction in fluorescence detected with flow cytometry. The VPD450 cell content analysis determined the percentage of cells responding to stimulation. The cells were stained with the following antibodies: anti-CD3-PerCP (Becton Dickinson, USA, #552851), anti-CD4-FITC (Becton Dickinson, USA, #555346), anti-CD8-APC-H7 (Becton Dickinson, USA, #560179) to identify T cells. Additional staining with PE-conjugated annexin V (Becton Dickinson, USA, #556421) or 7-amino actinomycin D (7-AAD) (Becton Dickinson, USA, #559925) facilitated the assessment of cell apoptosis. All analyses were performed using a BD FACSVerse™ Flow Cytometer (Becton Dickinson, USA). The results were analyzed with FlowJo software version 10.8.1 (Becton Dickinson, USA). The gating strategy for flow cytometry analyses is presented in **Supplementary Figure 1**.

### 2.7 Evaluation of cytokine/chemokine/growth factors in culture supernatants

The supernatants collected from 72h cultures of MP PBMC stimulated with CD3/CD28 mAb and exposed to the examined peptides were stored at -80°C until the test. For the evaluation of cytokines/chemokines/growth factors levels in the supernatants, Luminex^®^ xMAP^®^ technology was used. For the analysis, we used Human CD8+ T cell Magnetic Bead Panels (Merck Millipore, Germany, #HCD8MAG), and Human High Sensitivity T Cell Magnetic Bead Panel (Merck Millipore, Germany, # HISTMAG), which enables simultaneous quantification of the following: ITAC, GM-CSF, fractalkine, IFNγ, IL-10, MIP-3a, IL-12(p70), IL-13, IL-17A, IL-1β, IL-2, IL-21, IL-4, IL-23, IL-5, IL-6, IL-7, IL-8, MIP-1α, MIP- 1β, TNFα, sCD137, granzyme A, granzyme B, perforin, sFas, sFasL. The procedure was performed by the manufacturers’ instructions. Briefly, supernatants were incubated with a mixture of color-coded beads, pre-coated with analyte-specific capture antibodies. Next, a cocktail of biotinylated detection antibodies specific to the analyte of interest was added, followed by the addition of phycoerythrin (PE)-conjugated streptavidin, which binds to detection antibodies. The prepared samples were read by a Luminex MAGPIX^®^ Analyzer (Merck Millipore, Germany). Data were analyzed using xPONENT 4.2 software and presented as pg/ml. Data are presented only for analytes that reached the detection level set by the manufacturer.

### 2.8 T cell co-culture with SK-MEL-30 cell line

Magnetically isolated HD T cells were further processed for co-cultures with the SK-MEL-30 cell line. SK-MEL-30 cells and T cells, before plate seeding, were stained with 1 µl Violet Proliferation Dye 450 (VPD450) (Becton Dickinson, USA, #562158) for 10–15 min in the dark at 37°C following the manufacturer’s protocol and resuspended in a complete culture medium (RPMI 1640 culture medium supplemented with 10% FBS, 2 mM L-glutamine, 100 U/ml penicillin, and 100 μg/ml streptomycin). After staining, SK-MEL-30 cells were seeded at 0.5 × 10^6^ cells/well into a 6-well plate left to adhere. The T cells (2 × 10^6^ cells/well) were added to the SK-MEL-30 monolayer. The co-culture of these cells was stimulated with the ImmunoCult™ Human CD3/CD28 T Cell Activator in the presence of the examined peptides (**Table 1**) (25, 26). T cells incubated without stimulation comprised unstimulated control (CTRL(-)). Cells were incubated for 72 hours in standard culture conditions (37°C, 5% CO_2_). After incubation, T cells, as non-adherent cells, were collected in the supernatant, while SK-MEL-30 cells required trypsin-facilitated detachment. Specific staining of SK-MEL-30 cells was not required because of the adherent character of these cells. Subsequently, both types of cells were subjected to cytometric phenotypic analysis and evaluation of proliferation and apoptosis as described previously.

### 2.9 Statistical analysis

Statistical data analysis was performed using the GraphPad Prism program, version 9 (GraphPad Software, USA). The Kolmogorov-Smirnov and Lilliefors tests were used to test for normal distribution. Since data did not pass the normality tests, an appropriate nonparametric test for repeated measures (indicated in the Figure legends) was chosen with a significance level of p < 0.05. The comparison of parameters within the groups was evaluated with the ANOVA Friedman with Dunn’s *post hoc* test, between two paired measurements – with the Wilcoxon matched-pairs signed rank test and between two unpaired measurements – with the Mann-Whitney test. Unless otherwise stated, data are presented as medians and 25–75 quartile ranges.

## 3 Results

### 3.1 Patient characteristics

Following the assumed exclusion and inclusion criteria, we enrolled 10 immunotherapy-naive patients with good performance status (ECOG 0–1) after signing the informed consent forms. All patients had pathologically confirmed skin melanoma, one – locally advanced (stage IIIC) and others – disseminated disease (stage IV). The mean age of the patients group was 52.5 years; seven individuals harbor BRAF V600 mutation, of which one previously underwent BRAF/MEK targeted therapy. Evaluation of PD-L1 expression on melanoma cells is not a standard procedure in clinical practice yet – thus, it is not reported.

### 3.2 The influence of the examined peptides on the expression of BTLA and HVEM on T cells

**Figure 1:**
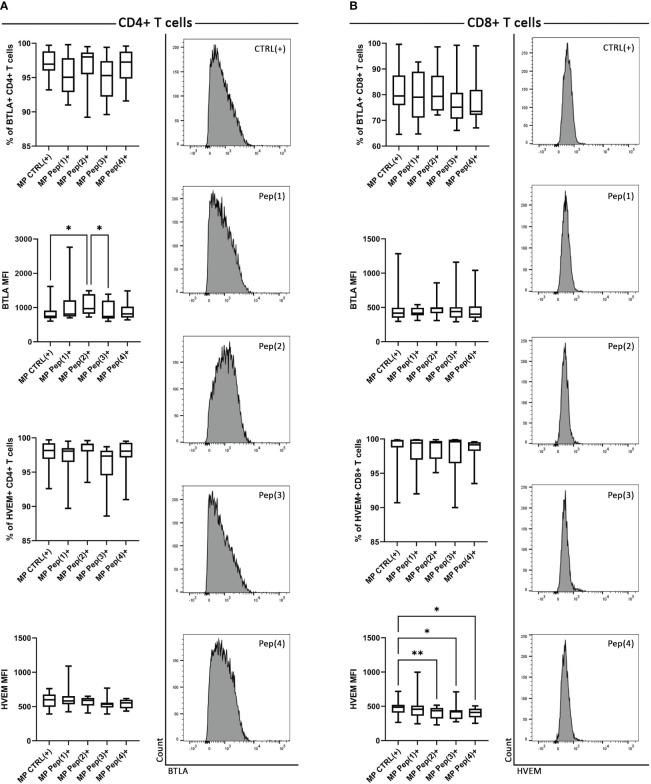
The expression of BTLA and HVEM on MP CD4+ T cells and MP CD8+ T cells. **(A)** shows MP CD4+ T cells, while **(B)** – MP CD8+ T cells. BTLA and HVEM expression is shown as a percentage of positive cells after 72h of cell culture in two variants: CTRL(+) – cells stimulated with CD3/CD28 mAb, Pep(x)(+) – stimulated cells exposed to the examined peptides: Pep(1)-Pep(4). The right panel of **(A)** represents exemplary histograms of BTLA expression on MP CD4+ T cells cultured with gD peptides. The right panel of **(B)** shows exemplary histograms of HVEM expression on MP CD4+ T cells cultured with gD peptides. Graphs represent median, percentiles and the maximum and minimum value of ten independent experiments; comparison between two paired measurements was performed with Wilcoxon matched-pairs rank test, between two unpaired measurements – with the Mann-Whitney test; *p < 0.05, **p < 0.01.

The analysis of MP CD4+ T cells showed slight, insignificant differences in the percentages of BTLA+ and HVEM+ cells when exposed to the examined peptides (**Figure 1A**). However, Pep(2) significantly increased the expression of BTLA, measured as median fluorescence intensity (MFI), compared to control – CTRL(+) (p=0.0041) and Pep(3) (p=0.0231). The addition of the examined peptides did not affect the percentages of MP HVEM+ CD4+ T or HVEM expression on these cells. By contrast, MP CD8+ downregulated the expression of HVEM upon exposure to the peptides, while the percentages of BTLA+ CD8+ cells or BTLA expression remained unchanged (**Figure 1B**). Pep(2), Pep(3) and Pep(4) led to an evident decrease in HVEM expression on CD8+ T cells, compared to CTRL(+) (p=0.0077, p=0.014 and p=0.0358, respectively).

### 3.3 gD peptides-associated activation of MP-derived T cells

**Figure 2:**
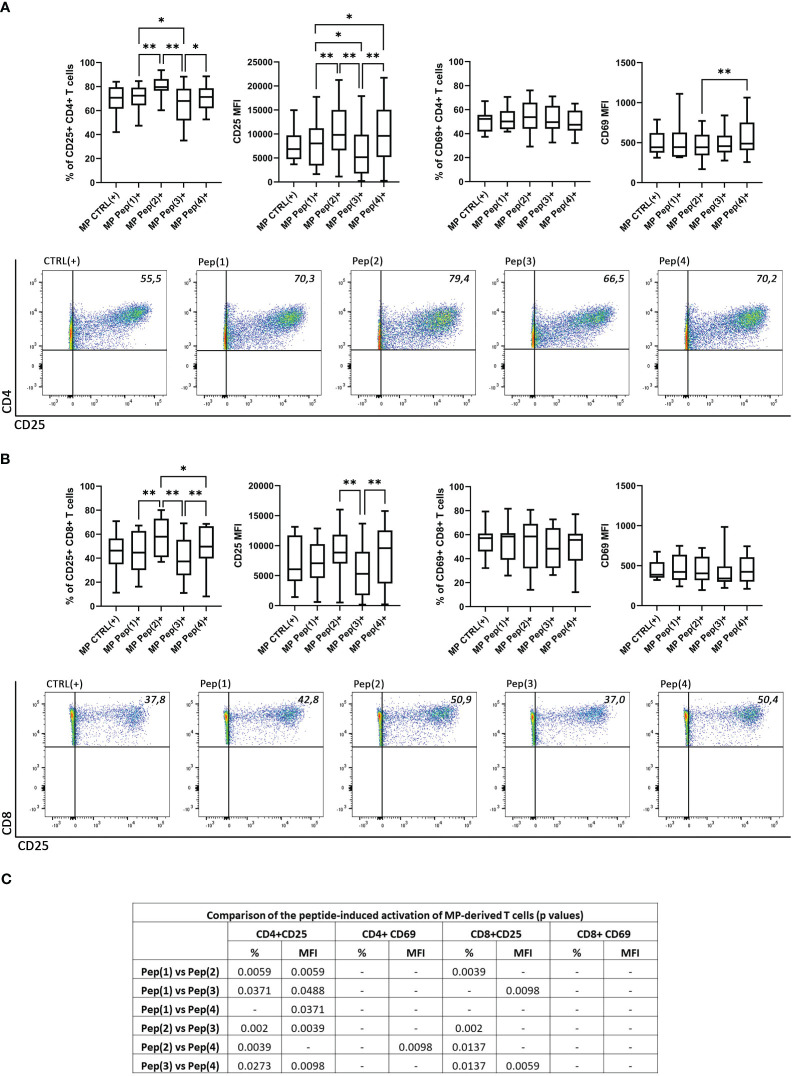
The expression of the activation markers on CD4+ and CD8+ MP T cells. **(A)** shows the percentage of CD25+ and CD69+ MP CD4+ T cells along with the exemplary dot plots representing CD25+ CD4+ T cells. **(B)** shows the percentage of CD25+ and CD69+ MP CD8+ T cells along with the exemplary dot plots representing CD25+ CD8+ T cells. **(C)** shows the comparison of the effects induced by the examined peptides (p values). The expression of CD25 and CD69 on T cell subpopulations is shown as a percentage of positive cells after 72h of cell culture in two variants: CTRL(+) – cells stimulated with CD3/CD28 mAb, Pep(x)(+) – stimulated cells exposed to the examined peptides: Pep(1)-Pep(4). Graphs represent median, percentiles and the maximum and minimum value of ten independent experiments; comparison between two paired measurements was performed with Wilcoxon matched-pairs rank test, between two unpaired measurements – with the Mann-Whitney test; *p < 0.05, **p < 0.01.

The activation of T cells was measured as changes in the expression of two activation markers: CD25 and CD69. MP CD4+ T cells changed the percentage of CD25+ cells and the surface expression of this activation marker in a peptide-dependent manner (**Figure 2A**). Pep(2) proved to be the most potent activation-inducing peptide, as it increased both the percentage of CD25+ CD4+ T cells and upregulated CD25 expression. Its effect surpassed two tested peptides, Pep(1) and Pep(3) (**Figure 2C**). By contrast, Pep(3) showed the weakest influence on CD25 expression in CD4+ T cells. Its effect measured as the percentage of CD25+ CD4+ T cells and CD25 expression was significantly lower compared to Pep(1), Pep(2), and Pep(4). The activation manner of the examined peptides was preserved in MP CD8+ T cells, highlighting the most promising effect of Pep(2) and the weakest influence of Pep(3) (**Figures 2B, C**). No differences in the percentages of CD69+ MP-derived T cells, both CD4+ and CD8+, or CD69 expression on these cells were detected with respect to the used stimulus (CD3/CD28 mAb or gD peptides) (**Figures 2A–C**).

### 3.4 T cell proliferation and apoptosis in MP-derived T cells exposed on gD-derived peptides

**Figure 3:**
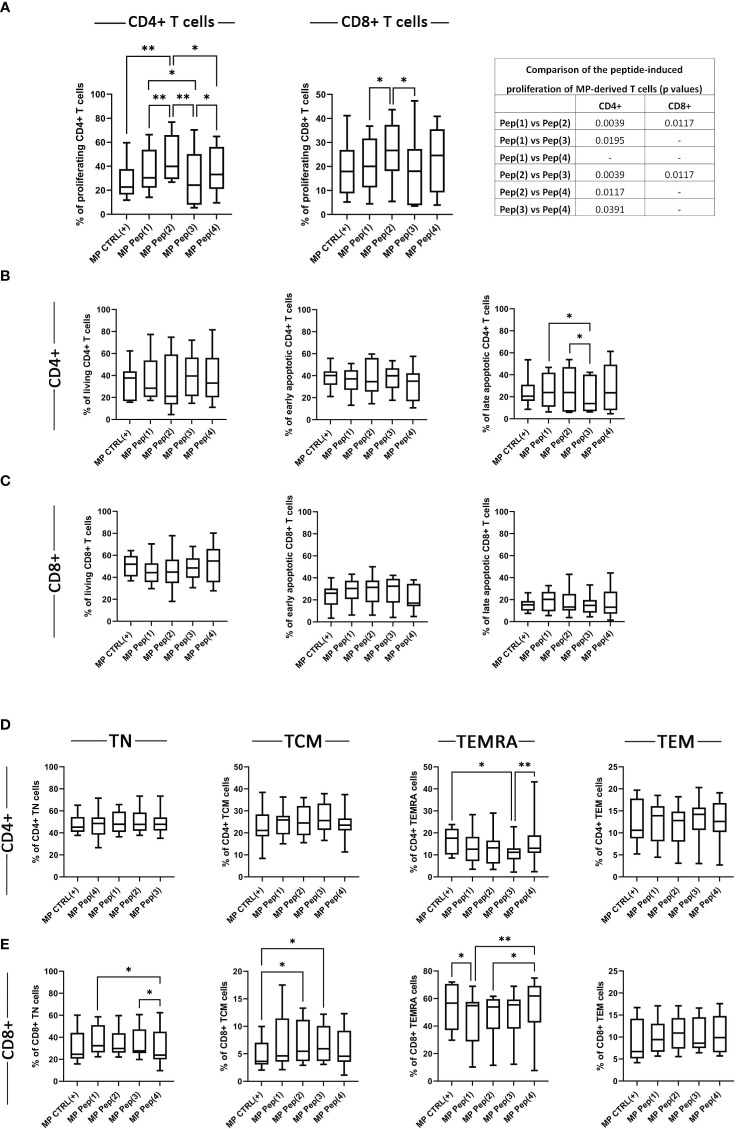
The influence of the examined peptides on MP T cells. Panel **(A)** shows the proliferation of MP CD4+ T cells, MP CD8+ T cells in tested culture variants: CTRL(+) – cells stimulated with CD3/CD28 mAb, Pep(x)(+) – stimulated cells exposed to the examined peptides: Pep(1)-Pep(4); and the comparison of the effects induced by the examined peptides (p values). Graphs in panels **(B, C)** represent the percentages of living, early apoptotic, late apoptotic and necrotic cells among MP CD4+ T cells **(B)** and MP CD8+ T cells **(C)**. T cell memory compartment analysis is depicted in panel **(D, E)**. **(D)** panel shows the percentages of: CD4+CD197+CD45RA- T cells (CD4+ TCM cells), CD4+CD197+CD45RA+ T cells (CD4+ TN cells), CD4+CD197-CD45RA+ T cells (CD4+ TEMRA cells), CD4+CD197-CD45RA- T cells (CD4+ TEM cells); while **(D)**: CD8+CD197+CD45RA- T cells (CD8+ TCM cells), CD8+CD197+CD45RA+ T cells (CD8+ TN cells), CD8+CD197-CD45RA+ T cells (CD8+ TEMRA cells), CD8+CD197-CD45RA- T cells (CD8+ TEM cells). Graphs represent median, percentiles and the maximum and minimum value of ten independent experiments; comparison between two paired measurements was performed with Wilcoxon matched-pairs rank test, between two unpaired measurements – with the Mann-Whitney test; *p < 0.05, **p < 0.01.

The proliferation of MP-derived stimulated T cells was evaluated with the dividing cell tracking (DCT) method, based on the VPD450 distribution to the daughter cells in every cell division. The examined peptides visibly increased the percentage of proliferating MP CD4+ T cells (**Figure 3A**). Pep(2) seemed to be the most potent compound that surpassed not only the control samples – CTRL(+), but also other peptides Pep(1), Pep(3) and Pep(4). Pep(3) was the least effective peptide, with little impact on MP CD4+ T cell proliferation. This peptide also proved less stimulating compared to Pep(2) regarding CD8+ T cell proliferation. Again, the pro-proliferative effect of Pep(2) on MP CD8+ T cells outranked other tested compounds (**Figure 3A**). Neither of the tested peptides impacted the percentages of living and early apoptotic CD4+ and CD8+ T cells, as well as late apoptotic CD8+ T cells (**Figures 3B, C**). The only differences were spotted in late apoptotic CD4+ T cells exposed to Pep(1), Pep(2), and Pep(3), with the lowest percentage of these cells in the presence of Pep(3) compared to Pep(1) (p=0.0488) and Pep(2) (p=0.0195) (**Figure 3B**).

### 3.5 The influence of gD-derived peptides on the T cell memory compartment in MPs

The evaluation of the potential influence of the examined peptides on the T cell memory compartment was performed via the assessment of the expression of CD197 and CD45RA. Based on the surface presence of these two markers the following memory subpopulations were distinguished: naïve T cells (TN): CD197+CD45RA+ T cells, central memory T cells (TCM): CD197+CD45RA-, effector memory T cells (TEM): CD197-CD45RA-, and effector memory cells re-expressing CD45RA (TEMRA): CD197-CD45RA+. The percentages of MP CD4+ TN, TCM, and TEM cells were comparable in all stimulation variants (**Figure 3D**). The examined compounds proved to affect the TEMRA compartment of MP CD4+ T cells. Pep(3) reduced the percentages of MP CD4+ TEMRA cells compared to MP CTRL(+) (p=0.0117) and Pep(4) (p=0.0039) (**Figure 3D**). Melanoma patient-derived CD8+ T cell memory compartment seemed more sensitive to the presence of the tested peptides (**Figure 3E**). The percentages of MP CD8+ TN cells decreased in the presence of Pep(4) compared to Pep(1) (p=0.0117) and Pep(3) (p=0.0391). Additionally, Pep(3), and Pep(2) increased the percentage of TCM CD8+ cells in relation to CTRL(+) (p=0.0146 and p=0.0185, respectively). The percentages of TEMRA CD8+ cells were significantly reduced by Pep(1) compared to CTRL(+) (p=0.0233) and Pep(4) (p=0.0078). The observed increasing effect of Pep(4) was also significant when referred to Pep(2) (p=0.0195).

### 3.6 The secretory profile of MP T cells exposed to the examined peptides

**Figure 4:**
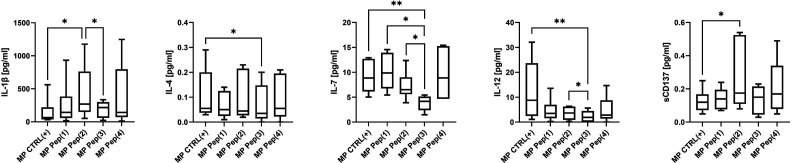
The secretory profile of stimulated MP T cells exposed to gD-derived peptides. Graphs show the secretion level of the selected cytokines: IL-1β, IL-4, IL-7, IL-12 and sCD137 evaluated in supernatants from tested culture variants: CTRL(+) – cells stimulated with CD3/CD28 mAb, Pep(x)(+) – stimulated cells exposed to the examined peptides: Pep(1)-Pep(4). Graphs represent median, percentiles and the maximum and minimum value of ten independent experiments. The comparison between two paired measurements was performed with Wilcoxon matched-pairs rank test, between two unpaired measurements – with the Mann-Whitney test; *p < 0.05, **p < 0.01.

A wide spectrum of cytokines/chemokines growth factors was evaluated using Luminex technology. Only several proteins were differently secreted by MP cells in response to the applied stimulus (**Figure 4**). The addition of the examined peptides resulted in augmented secretion of IL-1β. Pep(2) induced the most pronounced production of this cytokine compared to CTLR(+) (p=0.0436) and Pep(3) (p=0.0313). It also proved more effective in triggering sCD137 secretion than other examined compounds and CTRL(+) (p=0.0346). On the contrary, Pep(3) showed little impact on cytokines secretion compared to tested peptides. It significantly reduced levels of IL-4, IL-7 and IL-12 in relation to CTRL(+) (p=0.0215, p=0.006, p=0.0041, respectively). Additionally, the levels of IL-7 in Pep(3)-exposed cell cultures were significantly lower than in Pep(1)- or Pep(2)-cultured cells (p=0.0313 for both comparisons). IL-12 was also poorly released by Pep(3)-treated cells compared to other tested peptides, particularly to Pep(2) (p=0.0313).

### 3.7 The influence of gD-derived peptides on SK-MEL-30 cells in co-cultures with T cells from HDs

**Figure 5:**
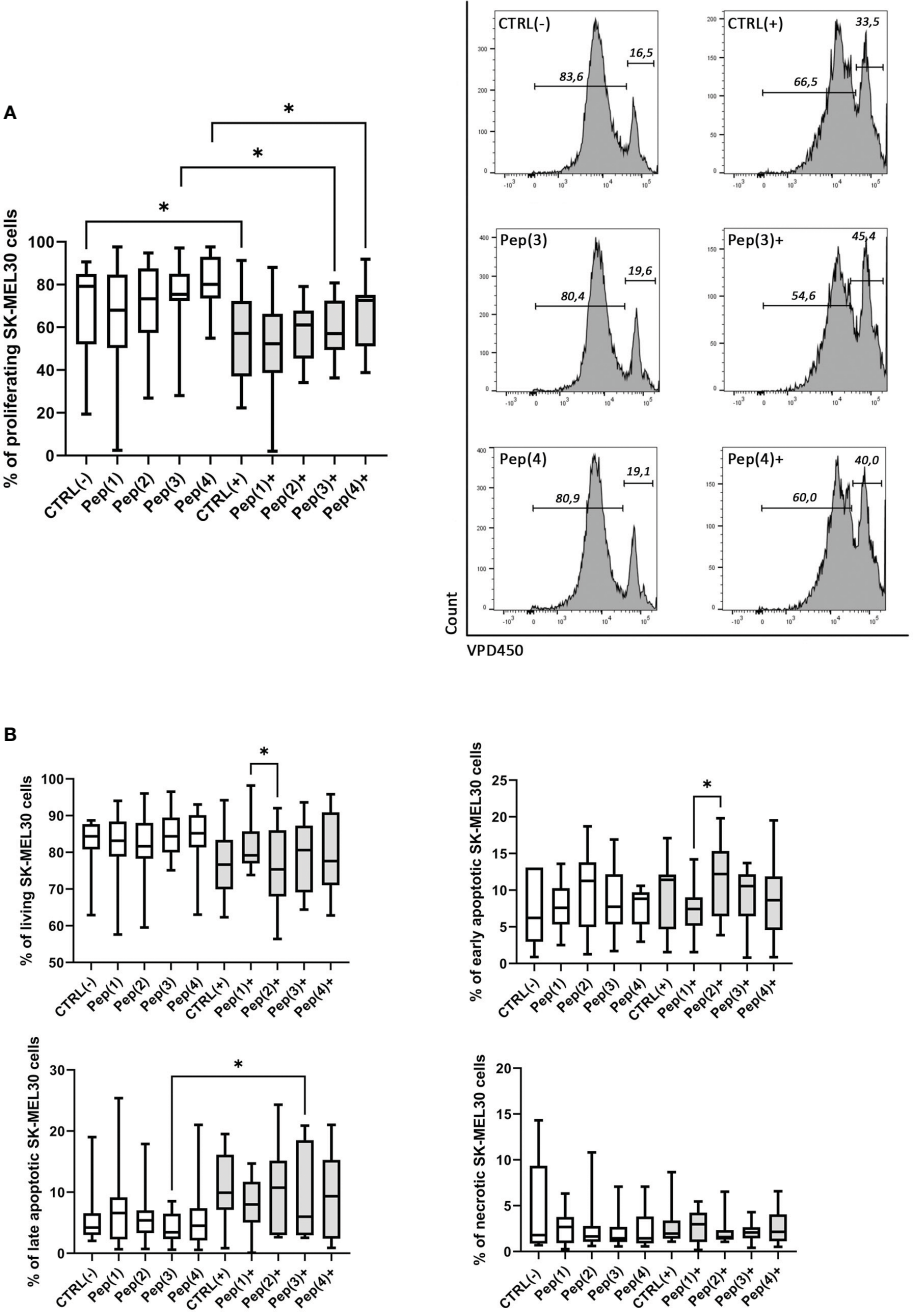
Proliferation and apoptosis of SK-MEL-30 cells co-cultured with T cells exposed to gD-derived peptides. Panel **(A)** shows the graph representing the percentage of proliferating SK-MEL-30 cells co-cultured with HD T cells for 72h in following conditions: CTRL(-) – unstimulated cells, Pep(x)(-) – cells exposed only to the examined peptides: Pep(1)-Pep(4), CTRL(+) – cells stimulated with CD3/CD28 mAb, Pep(x)(+) – stimulated cells exposed to the examined peptides: Pep(1)-Pep(4). The exemplary histograms are presented on the right, marker 1 (M1) indicates non-diving cells, M2 - the proportion of cells proliferating in response to the stimulus. Panel **(B)** represent the percentages of living, early apoptotic, late apoptotic and necrotic cells among SK-MEL-30 cells in tested co-culture variants. Graphs represent median, percentiles and the maximum and minimum value of ten independent experiments, comparison between two paired measurements was performed with Wilcoxon matched-pairs rank test, *p < 0.05.

SK-MEL-30 melanoma cell line was co-cultured with HD-derived T cells in two culture variants: (i) T cells were exposed only to the examined peptides, or (ii) T cells were simultaneously stimulated with CD3/CD28 mAb and exposed to the tested compounds. The unstimulated and unexposed to peptides T cells, called CTRL(-), did not exert any effect on SK-MEL-30 cells. Similar results were observed for unstimulated cells treated with the examined peptides only. When stimulated with CD3/CD28 mAb, T cells reduced the proliferation of SK-MEL-30 cells compared to unstimulated control (p=0.0273). The addition of gD peptides to stimulated T cells maintained this effect but with no significant differences between peptides (**Figure 5A**).

The observed reduction of SK-MEL-30 cell proliferation in co-cultures with stimulated and peptides-exposed T cells was accompanied by a decrease in the percentage of living melanoma cells (**Figure 5B**). Also, the proportions of late apoptotic melanoma cells varied depending on co-culture conditions, with a more pronounced effect upon the stimulation with the examined peptides. The significant differences between Pep(1) and Pep(2) focused mainly on the proportions between early apoptotic (p=0.00371) and living melanoma cells (p=0.0371). No evident changes or trends were detected in the necrotic or early apoptotic cell percentages, regardless of co-culture conditions.

## 4 Discussion

The concept of cancer immunosurveillance, coined by Burnet and Thomas over 50 years ago (31–33), focused on the immune system’s ability to recognize and eliminate nascent transformed cells. It created a background for further studies on the interplay between immune and cancer cells. The current theory of cancer immunoediting describes three phases of the immune system and tumor interaction, called the three Es: elimination, equilibrium, and escape (34). Eliminating transformed cells, present at the early stages of tumor formation, is a two-edged sword. It prevents cancer progression but also leads to the formation of less immunogenic and more immune-resistant tumor variants. These modified cells can survive the equilibrium phase and thrive in the third phase – the escape. The tumor-derived immune escape comprises a wide variety of specific mechanisms, including overexpression of ICPs (35). A deeper understanding of these processes may provide new therapeutic options.

HVEM overexpression is one of the immune escape mechanisms utilized by various tumor cells (36). Higher expression of HVEM on cancer cells and higher expression of BTLA on tumor-infiltrating lymphocytes (TILs) in metastatic melanoma patients was reported. The binding between BTLA and HVEM on two different cells is referred to as *trans* interaction, which promotes tumor evasion and impairs overall survival (13). However, HVEM is expressed not only on cancer cells but also on T cells and can interact with BTLA in *cis* conformation. This interaction was confirmed to deliver the co-inhibitory signal to T cell activation and proliferation (37). In the first step of our research, the expression of both proteins on CD4+ and CD8+ T cells was compared. Our data confirmed the differences in HVEM expression between MP and HD in T cells (**Supplementary Figure 2**). Higher percentages of HVEM+ CD4+ T cells and CD8+ T cells, as well as increased expression of HVEM on both subpopulations, were detected in patient samples. However, its inhibitory partner – BTLA, was visibly reduced in the analyzed cells. These data are contrary to the studies performed for the HCC patients, which showed that T cells from cancer patients persistently exhibited high levels of BTLA CD4+ but not CD8+ T cells compared to HD (8). The overexpression of BTLA was also observed in patients with melanoma (7), gallbladder cancer (38), diffuse large-B cell lymphoma (39), clear cell renal cell cancer (40), and prostate cancer (18). The studies analyzing HVEM expression on T cells are limited, but HCC patients showed that HVEM was downregulated on circulating CD8+ T cells but not CD4+ T cells compared to HD.

As mentioned previously, one of the ligands of HVEM is gD. It binds to HVEM at the same position as the BTLA protein and inhibits the formation of the BTLA-HVEM complex. The *N*-terminal fragment of the gD protein is involved in the interaction with HVEM, more precisely, the amino acid residues located at positions 7–15 and 24–30. The crystal structure of the HVEM-gD complex indicates that during protein binding, the conformation of the *N*-terminal fragment of the gD changes. In the native gD protein, the *N*-terminal fragment is disordered, whereas during interaction with HVEM, it adopts a β-harpin structure (24, 41). In our previous studies, we designed 15 peptides based on the *N*-terminal fragment of the gD protein as potential inhibitors of the BTLA-HVEM interaction. Four were chosen for further studies, displaying the best potential to block protein binding in ELISA and cellular assays. All of them have a disulfide bond in their structure, which was introduced to enable them to form a conformation similar to that which possesses the corresponding fragment of gD during interaction with HVEM. Therefore, the selected amino acid residues located precisely opposite each other in the structure of the gD were substituted for the cysteine residues (25, 26). Our previous studies have also shown that the presence and position of disulfide bonds impact the plasma stability of peptides. Numerous literature reports confirm these data (42–45); Pep(1) and Pep(2) have the highest stability in human plasma.

The next step of our studies was to evaluate the effect of gD-derived peptides on BTLA and HVEM expression on T cells. BTLA mediates inhibitory pathway during the binding with the CRD1 domain of HVEM. The anti-tumor immune response may be generated via the blockade of this region in HVEM. The gD-derived peptides examined in this study are targeted at the same domain in HVEM as BTLA. Therefore, they can block BTLA-HVEM interaction but do not interfere with the co-stimulatory HVEM-LIGHT pathway. The observed T cell reduction of BTLA expression upon exposure to gD peptides, especially by Pep(2), could be a hallmark of the altered immune response. The blocking of HVEM CRD1 by gD-derived peptides prevents interaction with BTLA and favors stimulatory signals provided by other HVEM ligands. Importantly, BTLA, during the binding to HVEM, delivers an inhibitory signal for T cells in both possible signaling modes: in *cis* and in *trans* (46). Therefore, the BTLA decrease, accompanied by the increased HVEM expression induced by the examined peptides, may promote T cell reactivation.

Melanoma, as a prototypic immunogenic tumor, significantly affects the activity of the immune system, leading to its profound dysfunction and exhaustion. Our previous study revealed that the initial activation status of MP T cells was higher than in HD (in press), suggesting a maintained ability to respond to dangerous signals. Unfortunately, the addition of stimulus (CD3/CD28 mAb) uncovered the exhausted state of MP-derived T cells (**Supplementary Figure 3**) and the narrower incitement range compared to HD T cells. Nevertheless, the examined peptides, especially Pep(2), proved beneficial in reviving MP T cell activity, measured as an increase in the activation markers expression. All tested peptides exerted their effect mainly on the late activation marker – CD25 in both analyzed CD4+ and CD8+ T cell subpopulations. Pep(2) not only boosted the activation of MP CD4+ T cells but visibly increased their proliferation rate. The activation level of MP CD8+ T cells, after exposure to Pep(2), did not reach the HD levels; however, it was significantly higher than MP CTRL(+). Obviously, the activation potential of immune cells varies between healthy individuals and cancer patients (**Supplementary Figures 3**, **4**), due to tumor-associated alterations.

A tumor microenvironment shaped by cancer cells utilizes a complex cytokine network to modify the activity of the immune system. Non-cancerous cells, such as immune cells, also contribute to TME via cytokine secretion (47). Depending on the repertoire of released cytokines, immune cells may either promote cancer progression and metastasis or induce anti-tumor response (48). The dual role of many cytokines in the TME and contradictory reports on their role in cancer progression make the analysis of the secretory profile of peptide-exposed MP T cells challenging. The exposure to the examined peptides affected the secretion of the following cytokines: IL-1β, sCD137, IL-4, IL-7, and IL-12, which were particularly noticeable for Pep(2) and Pep(3). Two analyzed cytokines, IL-1β and IL-4, are known for their pleiotropic effects in cancer (49, 50). IL-7 is mainly known for its anti-tumor effects mediated by immune-driven tumor eradication. Contrarily, the pro-tumor effect of IL-7 depends on the direct interaction of this cytokine with its receptor on cancer cells, thus promoting tumor growth (51). Due to the intricate cytokine network in cancer, it is difficult to determine the impact of gD-derived peptides on this precise immunological aspect. Anti-tumor cytotoxic cellular immune response activation is enhanced by IL-1β and sCD137 (52, 53). Pep(2) increased the release of sCD137, which provides a co-stimulatory signal for T cells (53). It may suggest that Pep(2) not only disrupts inhibitory BTLA-HVEM signaling but also may promote T cell activation. However, further studies on gD peptide-related cytokine secretion, are required to draw valid conclusions.

The stimulation of TCR may lead to several different outcomes, including programmed cell death. Apoptosis and necrosis contribute to the physiological contraction phase of the immune response. Additionally, strong stimulation may affect immune response because of the restimulation-induced death (54). The analysis of stimulated and peptide-exposed MP T cells confirmed that restimulation may increase immune cell apoptosis. The addition of the stimulus itself led to a decreased percentage of living MP CD4+ T cells compared to HD (**Supplementary Figure 4**), with no significant impact on MP CD8+ T cells. We speculate that the alterations in the percentage of apoptotic MP T cells are a consequence of intense stimulation and cell exhaustion rather than peptides themselves, as their biosafety has already been confirmed (**Supplementary Figure 4B**).

Our study revealed the subpopulation-specific alteration in the MP T cell memory compartment. The individual analysis of the influence of gD peptides on the MP T cells showed that CD4+ T cells are less prone to change their memory characteristics. Only Pep(3) caused a decrease in TEMRA CD4+ T cells from patients. On the contrary, MP CD8+ T cells responded more eagerly to the presence of gD peptides. The percentage of TN CD8+ T cells seemed to be peptide-dependent, as Pep(4) led to the most profound decrease of these cells. Pep(2) increased the percentages of MP TCM CD8+ T cells at the expense of TEMRA CD8+ T cells. Interestingly, the comparison of peptide-exposed MP and HD memory T cells showed reduced MP TN compartment of CD4+ and CD8+ cells, accompanied by increases in MP TEMRA cells (both CD4+ and CD8+) and MP CD4+ TEM cells. These results support the T cell activity reinforcement hypothesis via the gD peptide-mediated blockade of the BTLA-HVEM complex. This promising observation seems to be particularly important in relation to the deregulatory mechanisms of TME that promote the formation of dysfunctional lymphocytes (55).

To evaluate whether the examined gD-derived peptides can interfere with the BTLA-HVEM interaction, we established an *in vitro* co-culture model of fully immunocompetent healthy T cells and melanoma cell line. SK-MEL-30 melanoma cell line is used as a model cell line in a wide range of cancer-related studies (56, 57). Its high expression of both BTLA and HVEM was reported (58) and confirmed empirically (data not shown). The co-culture exposure only to gD peptides showed no significant impact on the proliferation of SK-MEL-30 cells. Our previous data also indicated that introducing the first signal, provided by CD3/CD28 mAb, was essential to elicit the immunomodulatory properties of gD peptides (27). We concluded that peptides themselves are insufficient to overcome cancer immune escape mechanisms and restore T cell anti-cancer activity. Therefore, the activating signal proved inevitable in inducing the effects caused by the disruption of the BTLA-HVEM complex by gD-derived peptides. Only the simultaneous addition of the activating stimulus and the examined peptides to co-cultures resulted in the reduced proliferation rate of the SK-MEL-30 cell line and higher percentages of apoptotic cells. Also, in this experiment, the most promising results were obtained for Pep(2).

The clinical introduction of ICI-based therapies revolutionized cancer immunotherapy. Monoclonal antibodies targeting inhibitory ICP proved useful, significantly increasing cancer patients’ overall survival and their quality of life. However, several drawbacks, including immune-related adverse events (irAEs), acquired immunotherapy resistance, or poor response (59, 60), limit their widespread use. Peptides and peptidomimetics seem attractive alternatives in ICP-related therapies (61). Peptides targeting ICPs present high selectivity and potency, good tolerance, predictable metabolism, and standardized synthesis protocols (62). Appropriate peptide formulation or peptide structure modification may rule out any potential disadvantages related to low bioavailability or short half-life. The presented study is in line with the worldwide trend in cancer immunotherapy, as it focuses on immunomodulation via the introduction of peptides disrupting inhibitory ICPs. gD-derived peptides, interacting with HVEM, can act dually. Through the interaction with the same binding site in HVEM as BTLA, they may block inhibitory signaling provided by BTLA. Additionally, this type of binding allows unrestricted interplay between HVEM and its co-stimulatory counterparts, LIGHT and LTα. The serum stability, target specificity, and inhibitory properties of the four tested gD-derived peptides were confirmed in our previous studies (25, 26). Here, we studied the ability of these peptides to interfere with BTLA-HVEM binding and facilitate CD3/CD28 mAb-mediated stimulation of MP T cells. Even though the response of MP immune cells did not reach this observed for HD samples, the exposure to gD peptides, especially Pep(2), proved beneficial in restoring the immune activity in patients. The differences between patient and healthy donors are the consequence of the initial, plausibly exhausted state of MP T cells. Therefore, we speculate that the Pep(2) may act as a trigger for overcoming T cell exhaustion in melanoma patients. It is believed that initial immune system disruption, resulting from tumor burden and cancer immune escape mechanism, is a key factor for a poor response rate of antibody-based ICP therapies (55). Therefore, the adjuvant and activating properties of Pep(2), reported in our study, may prove useful for future therapeutic strategies.

The peptides described above significantly differ in their immunomodulatory properties, although they are based on the same fragment of the gD binding to HVEM. The MM/GBSA analysis performed by us previously for the HVEM-gD complex allowed the identification of the amino acid residues in the gD protein that are crucial for the interaction with HVEM, and these are M11, A12, P14, N15, V24, Q27, L28, T29, P31, P32, and R35. T29 in Pep(1) and A12 in Pep(3) were substituted with cysteine residues, which could negatively impact their interaction with the target molecule. Pep(2) and Pep(4) have all the amino acid residues critical for binding to HVEM, so they were expected to show the most robust ability to block BTLA-HVEM interactions and affect T cells most effectively. In Pep(4), the cysteine residues that form the disulfide bond are located far apart, which may result in a more flexible structure of the peptide compared to Pep(2). Therefore, the peptide may not form a well-defined β-hairpin structure and fits the HVEM protein less closely (25, 26). The most promising peptide – Pep(2) – increased the percentage of activated CD4+ and CD8+ MP T cells. It also significantly promoted the proliferation rate of both tested MP T cell subpopulations. Additionally, it impacted the anti-tumor activity of healthy T cells in co-cultures with the SK-MEL-30 cell line, manifested via reduced proliferation of melanoma cells, decreased numbers of living tumor cells, and enhanced apoptosis. The opposite effects were reported for Pep(3), as it exerted the weakest effect on MP T cells. Pep(4) also affects T cells, although it is significantly weaker than observed for Pep(2). These differences are in agreement with our previous data (25, 26). The best ability to disrupt the BTLA-HVEM binding in both ELISA and the cellular assay was observed for the Pep(2). The obtained results indicate that the position of the disulfide bond is crucial for the inhibitory properties of the gD-derived peptides.

In concluding, the presented study paves a new path for the constant search for novel and effective cancer immunotherapies based on molecules interacting with ICPs. This paper reports the first group of peptide inhibitors effectively targeting HVEM that do not block the HVEM-LIGHT interaction. Particularly, Pep(2), when accompanied by the first signal, may help T cells regain their immunological functions. We made the first step in the long journey to the clinical application of peptide inhibitors in cancer immunotherapy. However, further research on the immunomodulating properties of gD-derived peptides is mandatory. Several aspects of these peptides’ mode of action and plausible clinical impact must be determined. BTLA and HVEM expression was confirmed on TILs and cancer cells in several types of tumors, including melanoma, hepatocellular carcinoma, colorectal cancer, glioblastoma, and others (63). Therefore, future studies need to include patients with various cancer types to evaluate the plausible correlation between peptides’ efficacy and type of malignancy. Besides, animal models are crucial in verifying the tested peptides’ long-term effects and their pharmacokinetic profile. Further modification of the structure of peptides based on the gD-binding fragment in order to obtain compounds that bind more strongly to HVEM also appears to be rational. This therapeutic approach targeting the HVEM protein seems justifiable in light of ongoing clinical trials evaluating monoclonal anti-BTLA in monotherapy or combined with other ICIs in various types of tumors (64). The gD-derived peptides, especially Pep(2), could be used in combination with these antibodies or with the other compounds targeting the BTLA protein. Provided we succeed in legitimizing the clinical application of the gD-derived peptide, it may significantly change the field of cancer management since these are the first non-antibody-based compounds targeting the HVEM protein. Incorporating such peptides into the currently applied immunotherapy regime may augment the efficacy of immunotherapies utilizing ICIs and may prevent severe side effects, including irAEs of antibodies-based therapy.

## Data availability statement

The raw data supporting the conclusions of this article will be made available by the authors, without undue reservation.

## Ethics statement

The studies involving humans were approved by Bioethics Committee for Scientific Research at the Medical University of Gdańsk. The studies were conducted in accordance with the local legislation and institutional requirements. The participants provided their written informed consent to participate in this study.

## Author contributions

KW: Formal analysis, Investigation, Writing – review & editing. KK: Investigation, Methodology, Writing – review & editing. JR: Investigation, Writing – original draft, Writing – review & editing. JJ: Writing – review & editing. AW: Conceptualization, Data curation, Formal analysis, Investigation, Methodology, Resources, Supervision, Validation, Visualization, Writing – original draft, Writing – review & editing. MS: Conceptualization, Data curation, Funding acquisition, Investigation, Project administration, Resources, Supervision, Writing – original draft, Writing – review & editing.
