# Supplementary material for: The effect of gD-derived peptides on T cell immune response mediated by BTLA-HVEM protein complex in melanoma patients
Source: Front Immunol. 2024 May 21;15:1362152. doi: 10.3389/fimmu.2024.1362152 (PMC11148245; doi:10.3389/fimmu.2024.1362152)
Supplement: Supplementary file 1 [file DataSheet_1.docx]

***Supplementary material***

The effect of gD-derived peptides on T cell immune response mediated by BTLA-HVEM protein The effect of gD-derived peptides on T cell immune response mediated by BTLA-HVEM protein complex in melanoma patients

Karolina Wojciechowicz^1^, Katarzyna Kuncewicz^2^, Jacek Rutkowski^3^, Jacek Jassem^3^, Anna Wardowska^1#^, Marta Spodzieja^2#^

^1^Department of Physiopathology, Faculty of Medicine, Medical University of Gdańsk, Poland

^2^Department of Biomedical Chemistry, Faculty of Chemistry, University of Gdańsk, Poland

^3^Department of Oncology and Radiotherapy, Medical University of Gdańsk, Poland

# - these authors equally supervised

*** Correspondence:**

Anna Wardowska, Department of Physiopathology, Faculty of Medicine, Medical University of Gdańsk, Poland; e-mail: [anna.wardowska@gumed.edu.pl](mailto:anna.wardowska@gumed.edu.pl);

Marta Spodzieja, Department of Biomedical Chemistry, Faculty of Chemistry, University of Gdańsk, Poland; e-mail: [marta.spodzieja@ug.edu.pl](mailto:marta.spodzieja@ug.edu.pl)

| **Supplementary Table 1. The characteristics of the examined peptide.** | | | | | | |
| --- | --- | --- | --- | --- | --- | --- |
| No. | Peptide name | Immune partner | Concentration used in  biological test  µg/ml | K_D_ [M] determined by SPR^1)^ | Blocking capacities towards BTLA/HVEM complex determined by cellular assay | Ref. |
| Pep (1) | gD(1-36)  (K10C-T29C) | HVEM | 100 | 1.30×10^-6^ | 45% ^2)^ | 25 |
| Pep (2) | gD(1-36)  (K10C-D30C) | HVEM | 100 | 4.36×10^-6^ | 56% ^2)^ | 26 |
| Pep (3) | gD(1-36)  (A12C-L25C) | HVEM | 250 | 9.35×10^-6^ | 37% ^2)^ | 26 |
| Pep (4) | gD(1-38)  (L4C-V37C) | HVEM | 250 | 9.53×10^-7^ (data not published) | 40% ^2)^ | 25 |

1. for the Pep(1-4) the affinity to HVEM protein was determined
2. The blocking capacities of Pep(1-4) were determined based on the percent of activation NF-κB – eGFP in the presence and absence of peptides [25,26]

| Supplemantary Table 2. Concentrations [pg/ml] of cytokines/chemokines/growth factors in T cell culture supernatants evaluated with Luminex® xMAP® technology | | | | | | | | | | |
| --- | --- | --- | --- | --- | --- | --- | --- | --- | --- | --- |
|  | **HEALTHY DONORS** | | | | | **MELANOMA PATIENTS** | | | | |
| CYTOKINES | **CTRL(+)**  Median  (min- max) | **Pep(1)**  Median  (min- max) | **Pep(2)**  Median  (min- max) | **Pep(3)**  Median  (min- max) | **Pep(4)**  Median  (min- max) | **CTRL(+)**  Median  (min- max) | **Pep(1)**  Median  (min- max) | **Pep(2)**  Median  (min- max) | **Pep(3)**  Median  (min- max) | **Pep(4)**  Median  (min- max) |
| GM-CSF | 3.8  (3.8-3.8) | 3.8  (3.8-3.8) | 3.8  (3.8-3.8) | 3.8  (3.8-3.8) | 3.8  (3.8-3.8) | 3.8 (3.8-3.8) | 3.8 (3.8-3.8) | 3.8 (3.8-3.8) | 3.8 (0.3-3.8) | 3.8 (3.8-3.8) |
| sCD137 | 0.190  (0.09-0.3) | 0.17  (0.13-0.2) | 0.24  (0.13-2.35) | 0.34  (0.15-2.35) | 0.19  (0.14-0.45) | 0.12 (0.05-0.25) | 0.14 (0.07-0.24) | 0.175 (0.08-0.54) | 0.15 (0.3-0.23) | 0.17 (0.05-0.49) |
| IFN-g | 1.21  (1.21-1.21) | 1.21  (1.21-1.21) | 1.21  (1.21-1.21) | 1.21  (1.21-1.21) | 1.21  (1.21-1.21) | 1.21 (1.21-1.21) | 1.21 (1.21-1.21) | 1.21 (1.21-1.21) | 1.21 (0.05-1.21) | 1.21 (0.04-1.21) |
| IL-1b | 5.04  (1.68-7.03) | 3.24  (1.74-7.51) | 5.91  (1.22-8.77) | 5.19  (1.97-10.7) | 3.42  (1.69-3.98) | 63.86 (31.31-560.12) | 143.69 (17.6-933.43) | 267.995 (52.21-1177) | 215.505 (21.3-333.94) | 142.17 (12.06-1248) |
| IL-2 | 1.9  (1.9-1.9) | 1.9  (1.9-1.9) | 1.9  (1.9-1.9) | 1.9  (1.9-1.9) | 1.9  (1.9-1.9) | 1.9 (1.9-1.9) | 1.9 (0.05-1.9) | 1.9 (0.09-1.9) | 1.14 (0-1.9) | 1.9 (0.02-1.9) |
| IL-4 | 0.42  (0.16-0.89) | 0.18  (0.11-0.26) | 0.2  (0.18-0.43) | 0.22  (0.12-0.33) | 0.2  (0.13-0.37) | 0.055 (0.03-0.29) | 0.05 (0.01-0.14) | 0.045 (0.02-0.23) | 0.035 (0-0.2) | 0.055 (0-0.21) |
| IL-5 | 0.1  (0.09-1.27) | 0.06  (0.05-0.14) | 0.08  (0.06-0.28) | 0.09  (0.06-0.33) | 0.07  (0.06-0.23) | 0.05 (0.03-0.18) | 0.04 (0.03-0.13) | 0.05 (0.04-0.59) | 0.04 (0-0.09) | 0.05 (0.0-0.53) |
| IL-6 | 0.38  (0.05-0.38) | 0.05  (0.03-0.3) | 0.14  (0.04-0.38) | 0.28  (0.13-0.38) | 0.12  (0.04-0.31) | 0.07 (0.02-0.38) | 0.07 (0.02-0.38) | 0.255 (0.03-0.38) | 0.25 (0.03-0.38) | 0.21 (0.01-0.38) |
| IL-7 | 5.97  (3.09-6.5) | 5.97  (4.28-10.97) | 8.21  (4.66-11.96) | 5.04  (3.89-14.46) | 7.88  (2.69-13.31) | 8.845 (5.04-12.91) | 9.855 (5.42-14.58) | 6.505 (3.89-12.38) | 4.18 (1.48-5.42) | 8.88 (4.66-15.44) |
| IL-8 | 909.54  (909.54-909.54) | 909.54 (909.54-909.54) | 909.54  (909.54-909.54) | 909.54 (909.54-909.54) | 909.54 (909.54-909.54) | 909.54 (494.48-909.54) | 909.54 (909.54-909.54) | 909.54 (909.54-909.54) | 909.54 (967.41-909.54) | 909.54 (909.54-909.54) |
| IL-10 | 5.07  (5.07-5.07) | 5.07  (5.07-5.07) | 5.07  (5.07-5.07) | 5.07  (5.07-5.07) | 5.07  (5.07-5.07) | 1.55 (0.07-5.07) | 1.32 (0.07-5.07) | 0.73 (0.07-5.07) | 0.66 (0.02-5.07) | 1.43 (0.08-5.07) |
| IL-12 | 0.44  (0.22-2.04) | 0.22  (0.19-0.45) | 0.31  (0-0.63) | 0.3  (0-0.54) | 0.19  (0-0.36) | 8.785 (1.12-  32.08) | 3.475 (0.26-13.57) | 3.67 (0.33-6.38) | 1.905 (0-5.66) | 2.835 (0-14.69) |
| IL-13 | 1.91 (1.91-1.91) | 1.91 (1.82-1.91) | 1.91 (1.91-1.91) | 1.91 (1.78-1.91) | 1.91 (1.88-1.91) | 1.225 (0.33-1.91) | 1.495 (0.31-1.91) | 1.895 (1.08-1.91) | 1.045 (0-1.91) | 1.73 (0.01-1.91) |
| IL-17a | 3118 (2200-3118) | 3118 (2236-3118) | 3118 (3118-3118) | 3118 (2146-3118) | 3118 (2866-3118) | 1243.765 (93.19-3118) | 1046.37 (78.08-3118) | 769.555 (148.02-3118) | 961.39 (11.25-2799) | 1182.5 (66.77-3118) |
| IL-21 | 325 (232.54-1150) | 71.37 (34.67-183.37) | 216.37 (90.72-1169) | 169.36 (61.71-503.31) | 71.13 (27.89-607.99) | 489 (17.24-1169) | 26.635 (1-1169) | 35.88 (3.36-1169) | 21.825 (0.25-318.03) | 32.625 (0.45-905.1) |
| IL-23 | 9.25 (7.8-13.98) | 7.8 (7.8-12.35) | 9.25 (6.43-19.14) | 7.8 (7.8-13.98) | 9.25 (7.8-10) | 16.52 (6.43-22.75) | 15.27 (9.25-20.93) | 14.82 (6.43-24.59) | 12.375 (6.43-19.14) | 14.395 (8.52-32.21) |
| MIP-1a | 0.77 (0.77-0.77) | 0.77 (0.77-0.77) | 0.77 (0.77-0.77) | 0.77 (0.77-0.77) | 0.77 (0.77-0.77) | 0.77 (0.77-0.77) | 0.77 (0.77-0.77) | 0.77 (0.03-0.77) | 0.77 (0.77-0.77) | 0.77 (0.03-0.77) |
| MIP-1b | 1.77 (1.77-1.77) | 1.77 (1.77-1.77) | 1.77 (1.77-1.77) | 1.77 (1.77-1.77) | 1.77 (1.77-1.77) | 1.77 (1.77-1.77) | 1.77 (1.7-1.77) | 1.77 (1.77-1.77) | 1.77 (0.24-1.77) | 1.77 (0.22-1.77) |
| MIP-3a | 2024 (689.6-3200) | 1424 (840.32-2731) | 2026 (1168-3200) | 3200 (866.78-3200) | 2355 (1027-2780) | 888.445 (210.12-3200) | 1026.15 (310.03-3011) | 602.27 (376.38-2294) | 781.755 (24.83-3200) | 984.855 (99.09-2731) |
| TNF-a | 0.51 (0.51-0.51) | 0.51 (0.51-0.51) | 0.51 (0.51-0.51) | 0.51 (0.51-0.51) | 0.51 (0.51-0.51) | 0.51 (0.51-0.51) | 0.51 (0.51-0.51) | 0.51 (0.51-0.51) | 0.51 (0.03-0.51) | 0.51 (0.02-0.51) |
| Granzyme A | 2.44 (1.98-19.35) | 1.99 (1.8-5.09) | 2.82 (1.86-7.2) | 3005 (2.01-4.98) | 3.15 (1.93-5.6) | 3.0 (0.49-19.35) | 2.125 (0.42-3.6) | 3.24 (0.65-19.35) | 2.085 (0.17-5.11) | 3.15 (0.26-19.35) |
| Granzyme B | 1.27 (1.27-1.27) | 1.27 (1.27-1.27) | 1.27 (1.27-1.27) | 1.27 (1.27-1.27) | 1.27 (1.27-1.27) | 1.27 (1.04-1.27) | 1.27 (0.29-1.27) | 1.27 (1.27-1.27) | 0.885 (0.03-1.27) | 1.27 (0.04-1.27) |
| Perforin | 0.6 (0.46-0.89) | 0.53 (0.43-0.54) | 0.63 (0.42-0.74) | 0.7 (0.48-0.8) | 0.67 (0.46-0.68) | 0.715 (0.33-0.87) | 0.52 (0.31-0.73) | 0.7 (0.4-0.78) | 0.47 (0.28-0.74) | 0.67 (0.32-0.78) |
| sFas | 1.25 (1.25-1.47) | 1.25 (1.25-1.25) | 1.25 (1.25-1.64) | 1.25 (1.25-1.47) | 1.25 (1.25-1.66) | 1.25 (1.25-1.25) | 1.25 (1.25-1.25) | 1.25 (1.25-1.25) | 1.25 (1.25-1.25) | 1.25 (1.25-1.25) |
| sFasL | 0.34 (0.29-0.38) | 0.29 (0.24-0.33) | 0.33 (0.3-0.4) | 0.37 (0.32-0.47) | 0.34 (0.3-0.636) | 0.255 (0.1-0.33) | 0.205 (0.1-0.36) | 0.26 (0.13-0.33) | 0.195 (0.1-0.34) | 0.29 (0.1-0.33) |
| ITAC | 27.08 (17.2-44.81) | 27.29 (13.8-37.04) | 33.05 (13.37-68.25) | 16.02 (12.93-75.23) | 17.84 (10.78-45.22) | 197.57 (29.97-1078) | 125.865 (83.62-555.6) | 121.7 (78.02-389.23) | 119.56 (56.25-408.33) | 366.455 (39.25-955.92) |
| Fractalkine | 7.33 (7.33-7.33) | 7.33 (7.33-7.33) | 7.33 (7.33-7.33) | 7.33 (7.33-7.33) | 7.33 (7.33-7.33) | 7.33 (7.33-7.33) | 7.33 (7.33-7.33) | 7.33 (7.33-7.33) | 7.33 (7.33-7.33) | 7.33 (7.33-7.33) |


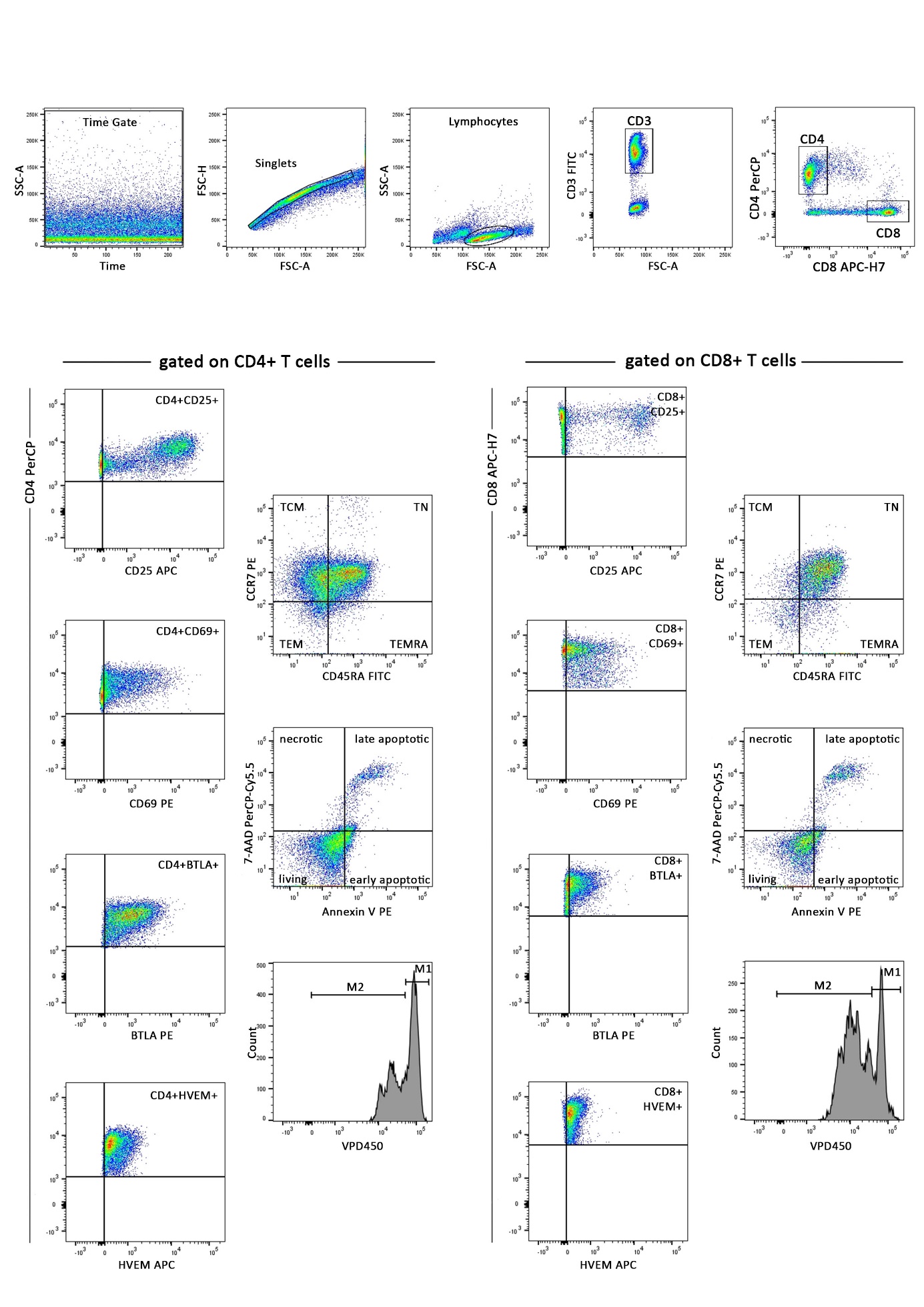


**Supplementary Figure 1. Gating strategy.** Twenty thousand events corresponding to the light scatter characteristics of viable lymphocytes were acquired from each sample in order to analyze lymphocyte subpopulations in vitro, thirty thousand events - in order to perform the dividing cell tracking (DCT) method. Firstly, a time gate was applied with the following doublet exclusion. Then, lymphocytes were selected based on forward (FSC) and side scatter (SSC) characteristics and their positivity for surface antigens (CD3, CD4, CD8, CD25, CD69, HVEM, etc.). Cytometric data were analyzed with FlowJo 10 software (Beckton Dickinson, USA). The graph shows the representative dot plots and histograms for CD4+ T cells (left panel) and CD8+ T cells (right panel). Lymphocytes were selected based on FCS (forward side scatter) and SSC (side scatter characteristics). T cells were subsequently distinguished based on their expression of CD3+. Then T cell subpopulations were identified based on their positivity for CD4 and CD8 antigen. Next, subpopulations expressing activation markers: CD25 and CD69 were identified. Then, CD4+ T cells/CD8+ T cells positive for either BTLA or HVEM were distinguished. Memory CD4+/CD8+ T cell compartments were analyzed based on the surface expression of CCR7 (CD197) and CD45RA. The following phenotypic classification of T cells was used: CD45RA+CCR7+ naive (TN), CD45RA-CCR7+ central memory (TCM), CD45RA-CCR7- effector memory (TEM), and CD45RA+CCR7- effector memory re-expressing CD45RA (TEMRA) T cells. The annexin V and 7-AAD staining was used to distinguish between viable cells (annexin V and 7-AAD negative), cells in early apoptosis (annexin V positive and 7-AAD negative), in late apoptosis (positive for both annexin V and 7-AAD), and necrotic cells (cells that are only 7-AA positive). Finally, a proliferation of CD4+/CD8+ T cells was analyzed (J) - marker 1 (M1) indicate non-diving cells, M2 - the proportion of cells proliferating in response to stimulation.


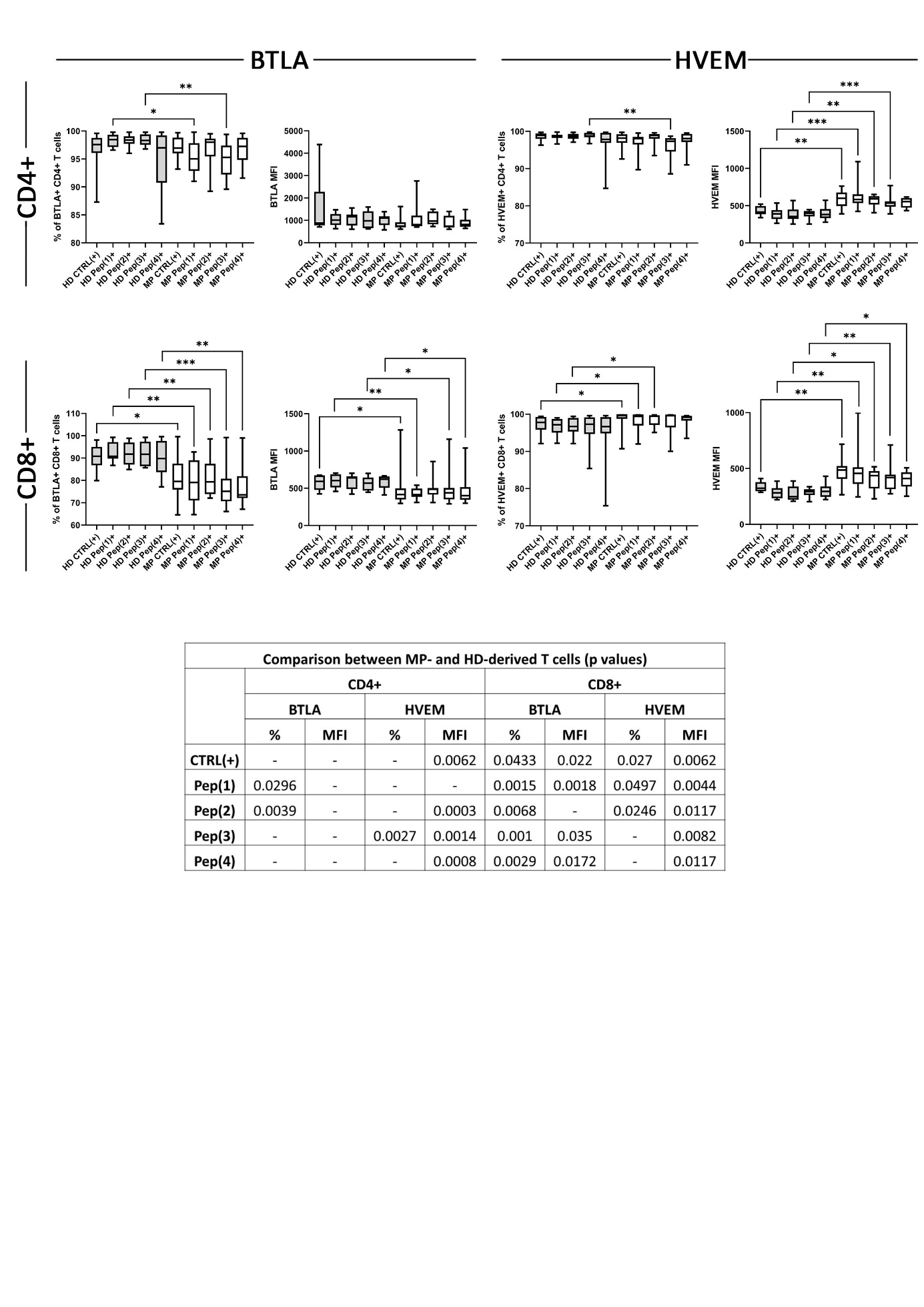


**Supplementary Figure 2. The expression of BTLA and HVEM on CD4+ T cells and CD8+ T cells from MP and HD.** The upper panel shows CD4+ T cells, while the middle one – CD8+ T cells, table depicts comparison between MP- and HD-derived T cells (p values). BTLA and HVEM expression is shown as a percentage of positive cells after 72 of cell culture in the following conditions: CTRL(+) – cells stimulated with CD3/CD28 mAb, Pep(x)(+) – stimulated cells exposed to the examined peptides: Pep(1)-Pep(4). Graphs represent median, percentiles and the maximum and minimum value of ten independent experiments per group (HD/MP); comparison between two paired measurements was performed with Wilcoxon matched-pairs rank test, between two unpaired measurements – with the Mann-Whitney test; * p < 0.05, ** p < 0.01, *** p < 0.001.


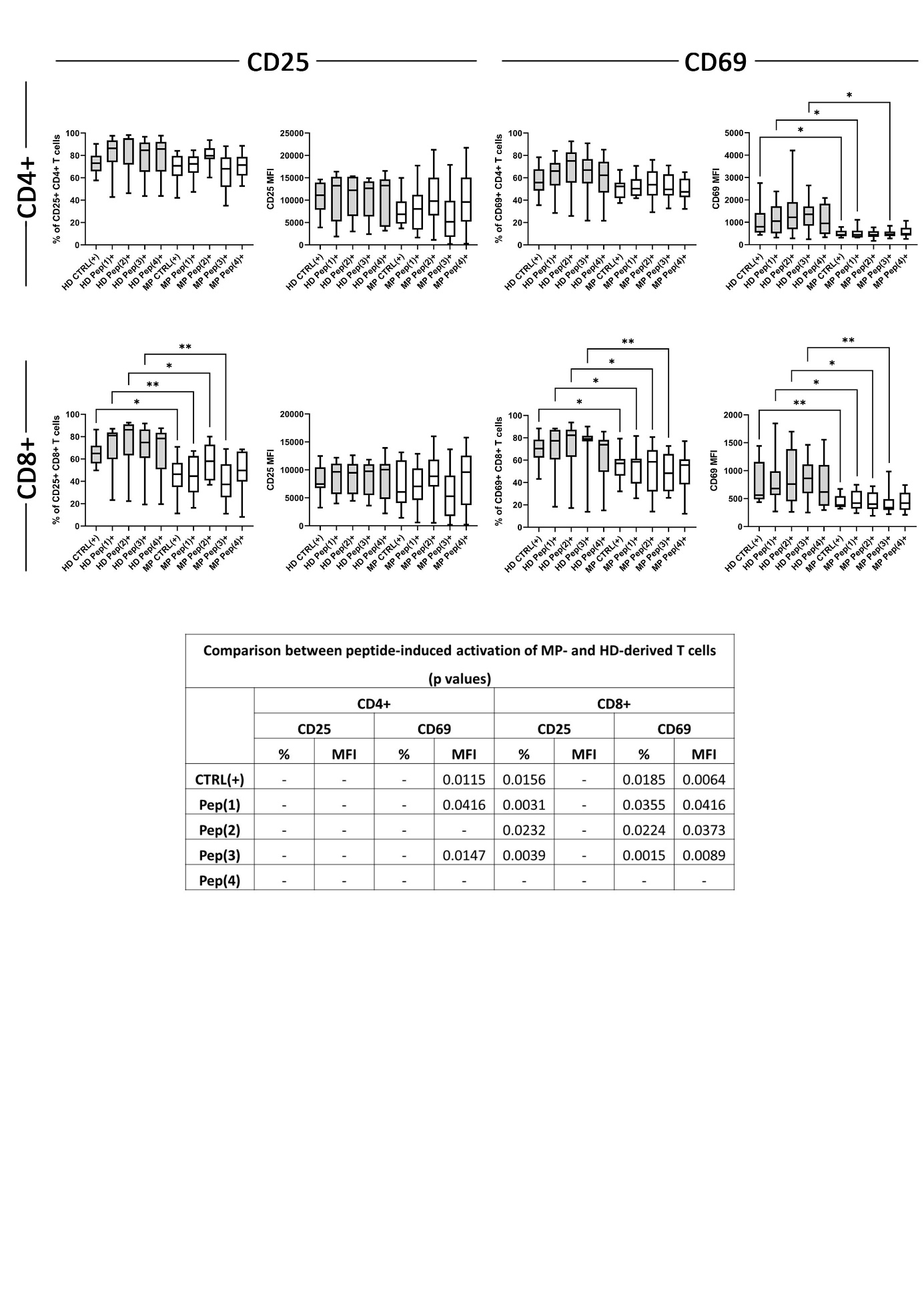


**Supplementary Figure 3. The expression of the activation markers on CD4+ and CD8+ T cells.** The upper panel shows CD4+ T cells, while the middle one – CD8+ T cells, table depicts comparison between MP- and HD-derived T cells (p values). The expression of CD25 and CD69 on T cell subpopulations from HD and MP is shown as a percentage of positive cells after 72h of cell culture in the following conditions: CTRL(+) – cells stimulated with CD3/CD28 mAb, Pep(x)(+) – stimulated cells exposed to the examined peptides: Pep(1)-Pep(4). Graphs represent median, percentiles and the maximum and minimum value of ten independent experiments per group (HD/MP), comparison between two paired measurements was performed with Wilcoxon matched-pairs rank test, between two unpaired measurements – with the Mann-Whitney test; * p < 0.05, ** p < 0.01.


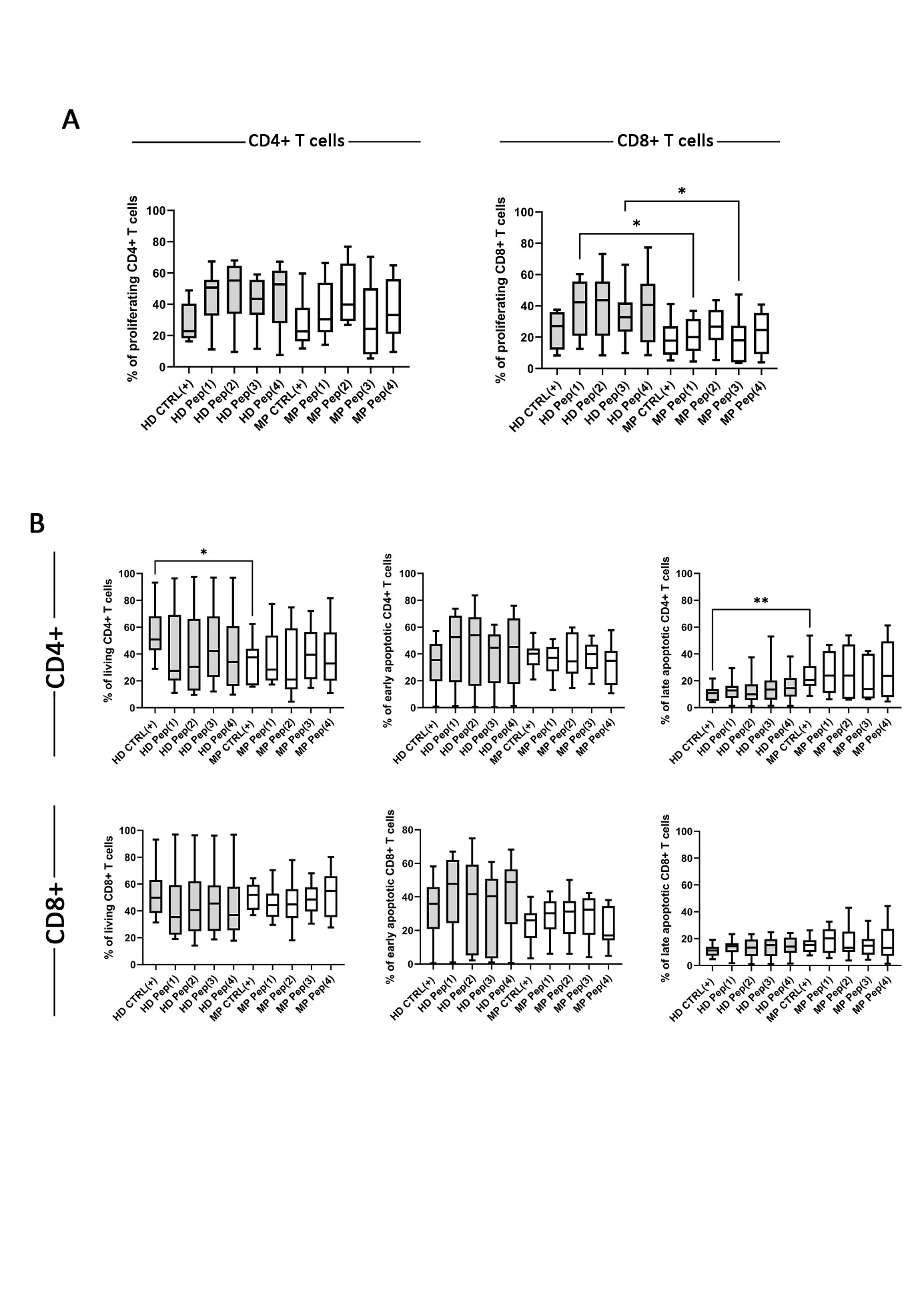


**Supplementary Figure 4. The proliferation and apoptosis of T cells cultured with or without** **Pep(1)-Pep(4).** Panel (A) shows the proliferation of HD and MP CD4+ and CD8+ T cells from two culture conditions: CTRL(+) – cells stimulated with CD3/CD28 mAb, Pep(x)(+) – stimulated cells exposed to the examined peptides: Pep(1)-Pep(4). Panel (B) represent the percentages of living, early apoptotic, late apoptotic and necrotic cells among CD4+ T cells (upper) and CD8+ T cells (lower) in tested culture variants. Graphs represent median, percentiles and the maximum and minimum value of ten independent experiments per group (HD/MP); comparison between two paired measurements was performed with Wilcoxon matched-pairs rank test, between two unpaired measurements – with the Mann-Whitney test; * p < 0.05, ** p < 0.01.


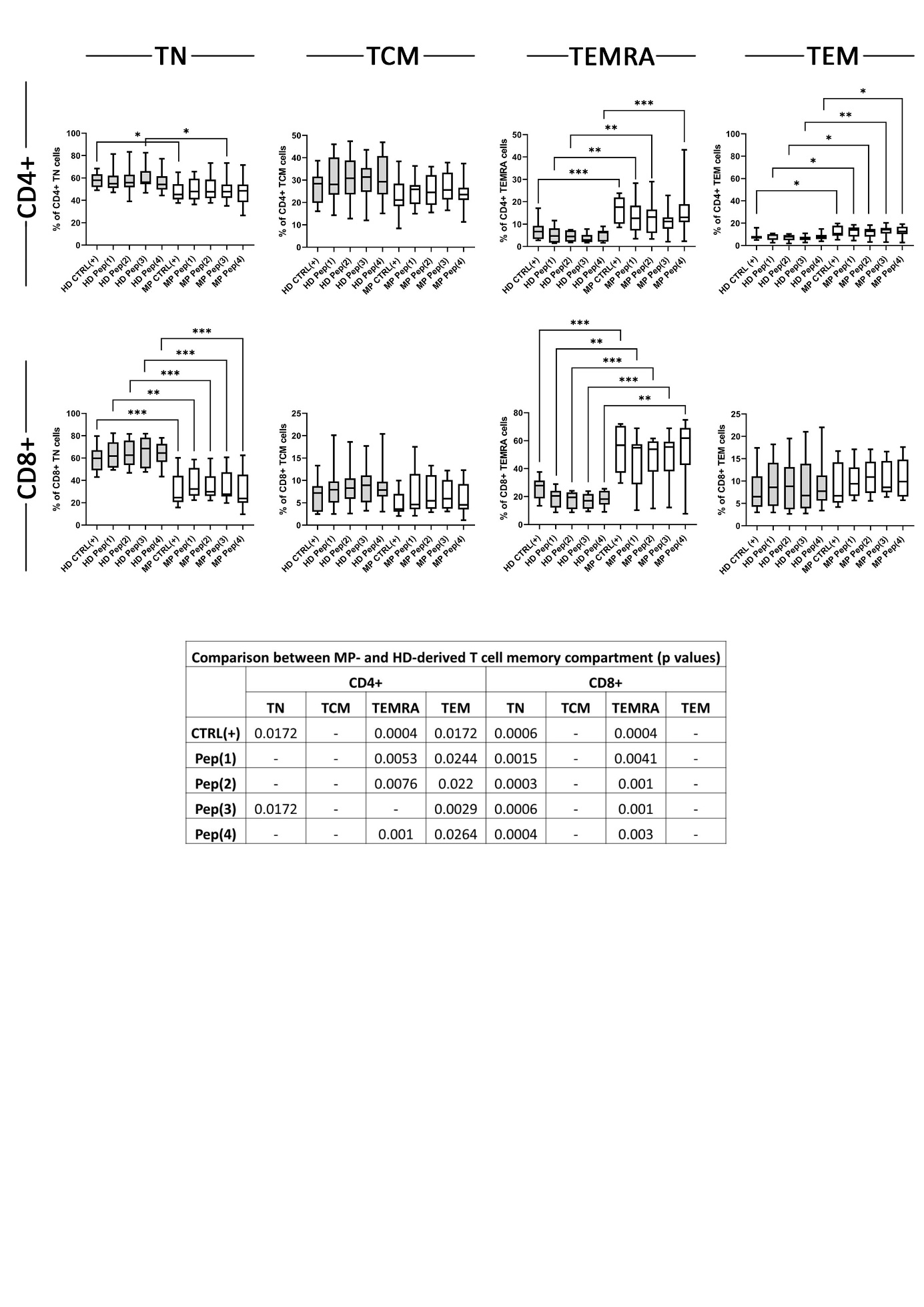


**Supplementary Figure 5.** **The distribution of T cell memory subpopulations in response to Pep(1)-Pep(4) exposure.** The upper panel shows CD4+ T cell memory compartment, while the middle one – CD8+ memory T cells, table depicts comparison between MP- and HD-derived T cells (p values). Following subpopulations are presented on the graphs: CD4+CD45RA-CCR7+ T cells (CD4+ TCM cells), CD4+CD45RA+CCR7+ T cells (CD4+ TN cells), CD4+CD45RA+CCR7- T cells (CD4+ TEMRA cells), CD4+CD45RA-CCR7- T cells (CD4+ TEM cells); CD8+CD45RA-CCR7+ T cells (CD8+ TCM cells), CD8+CD45RA+CCR7+ T cells (CD8+ TN cells), CD8+ CD45RA+CCR7- T cells (CD8+ TEMRA cells), CD8+ CD45RA-CCR7- T cells (CD8+ TEM cells). Graphs represent median, percentiles and the maximum and minimum value of ten independent experiments per group (HD/MP); comparison between two paired measurements was performed with Wilcoxon matched-pairs rank test, between two unpaired measurements – with the Mann-Whitney test; * p < 0.05, ** p < 0.01, *** p < 0.001.
